# *Aloysia deserticola* (rica-rica) essential oil impairs inhibitory synaptic transmission in hippocampal CA1 pyramidal neurons

**DOI:** 10.3389/fphar.2026.1917739

**Published:** 2026-10-14

**Authors:** Juan Ahumada, Pablo Morel-Alonso, Nicole Vidal-Herrera, Koyam Morales-Weil, Waleska Vera, Marco Fuenzalida

**Affiliations:** 1 Centro de Neurobiología y Fisiopatología Integrativa (CENFI), Laboratorio de Neuroplasticidad, Instituto de Fisiología, Facultad de Ciencias, Universidad de Valparaíso, Valparaíso, Chile; 2 Millennium Nucleus of Neuroepigenetics and Plasticity (EpiNeuro), Santiago, Chile; 3 Centro Interdisciplinario de Neurociencia de Valparaíso (CINV), Facultad de Ciencias, Universidad de Valparaíso, Valparaíso, Chile; 4 Facultad de Farmacia, Escuela de Química y Farmacia, Laboratorio de Química de Metabolitos Bioactivos Universidad de Valparaíso, Valparaíso, Chile; 5 Programa de Doctorado en Ciencias, Mención Neurociencia, Universidad de Valparaíso, Valparaíso, Chile; 6 Facultad de Ciencias de la Vida, Universidad Viña del Mar, Viña del Mar, Chile; 7 Centro de Investigación, Desarrollo e Innovación de Productos Bioactivos (CInBIO), Universidad de Valparaíso, Valparaíso, Chile

**Keywords:** *Aloysia deserticola*, essential oil, GABA_A_ receptor, hippocampal CA1, inhibitory synaptic transmission, patch-clamp electrophysiology, thujone

## Abstract

*Aloysia deserticola* is an aromatic plant native to the Chilean Andean highlands that has traditionally been used in herbal preparations. Although a previous behavioral study reported the anxiolytic and antidepressant-like effects of rica-rica essential oil in an animal model, its neurophysiological actions at cellular and synaptic levels remain largely unexplored. In this study, we characterized *A. deserticola* essential oil (AD-EO) using gas chromatography–mass spectrometry (GC–MS) and examined its acute effects on GABAergic synaptic transmission via whole-cell patch-clamp recordings from CA1 pyramidal neurons in acute dorsal hippocampal slices obtained from C57BL/6 J mice. GC–MS analysis revealed an enriched thujone chemical profile, with peaks tentatively identified as α- and β-thujone accounting for 89.06% and 3.38% of the total chromatographic area, respectively. Application of AD-EO to the bath reduced evoked inhibitory postsynaptic current (eIPSC) amplitude in a concentration-dependent manner and shortened eIPSC decay kinetics. This is consistent with a postsynaptic contribution to the suppression of inhibitory signaling. At intermediate-to-high estimated thujone-equivalent concentrations, AD-EO increased the paired-pulse ratio. Coefficient-of-variation analysis supported the presence of an additional presynaptic component. Consistent with these findings, AD-EO reduced the frequency and amplitude of miniature inhibitory postsynaptic currents. Furthermore, the effects on eIPSC amplitude and the paired-pulse ratio were reversible following washout. Together, these results demonstrate that AD-EO suppresses inhibitory synapses in CA1 pyramidal neurons via effects consistent with pre- and postsynaptic *locus*. These results provide electrophysiological evidence that AD-EO modulates hippocampal inhibitory transmission and support further investigation of the mechanisms underlying the neurophysiological actions of this complex natural preparation.

## 1 Introduction

*Aloysia deserticola* (Phil.) Lu-Irving and O’Leary (*A. deserticola*), commonly known as rica-rica and formerly classified as *Acantholippia deserticola* (Lu-Irving et al., 2014), is an aromatic species of the Andean highlands of northern Chile. It has traditionally been used as an herbal infusion in folk medicine to treat gastrointestinal and inflammatory conditions and urinary tract disorders (Baraona et al., 1958; Villagrán et al., 2003; Larrazabal-Fuentes et al., 2019; Cordero et al., 2022). Phytochemical studies have identified a complex essential-oil profile rich in bioactive monoterpenes and other volatile compounds. Several natural terpenoids have been shown to interact with components of the GABAergic system (Manayi et al., 2016). Separately, *A. deserticola* essential oil (AD-EO) has been reported to produce antidepressant- and anxiolytic-like effects in female rats (Benites et al., 2013), indicating that the preparation can influence CNS-related behavioral outcomes. However, the cellular and synaptic mechanisms underlying the neurophysiological actions of AD-EO remain largely unexplored. A distinctive feature of AD-EO is its thujone-enriched chemotype (Rojo et al., 2006; Ortiz et al., 2019; Larrazabal-Fuentes et al., 2019). Thujone monoterpenes, particularly α- and β-thujone, have neuroactive effects primarily through modulation of GABA_A_ receptor signaling. Studies in recombinant GABA_A_ receptors and cultured hippocampal neurons show that α-thujone inhibits GABA_A_ receptor-mediated currents and alters GABAergic transmission (Höld et al., 2000; Szczot et al., 2012; Czyzewska and Mozrzymas, 2013). This is particularly relevant in the hippocampus, where GABAergic inhibition regulates CA1 pyramidal neuron activity and maintains excitation/inhibition balance. Thus, thujone-rich natural products may influence hippocampal function by altering GABAergic synaptic control. In the present study, we used a combination of chemical profiling and whole-cell patch-clamp electrophysiology to examine the effects of AD-EO on inhibitory synaptic transmission in the dorsal hippocampus of mice. Using gas chromatography–mass spectrometry (GC–MS), we characterized AD-EO and evaluated its effects on evoked inhibitory postsynaptic currents (eIPSCs) recorded from CA1 pyramidal neurons in acute hippocampal slices. Our results demonstrate that AD-EO significantly modulates GABAergic synaptic transmission in hippocampal circuits involved in cognitive processing. This provides new insight into the neurophysiological actions of this native medicinal plant.

## 2 Methods

### 2.1 Collection, identification, and extraction

The aerial parts of *A*. *deserticola* were collected in August 2023 near the town of Río Grande (22°40’08.4”S 68°18’30.5”W), at 3,200 m.a.s.l., Region of Antofagasta, Chile. The identified plant corresponds to a specimen deposited in the Herbarium of the University of Playa Ancha VALPL (accession No 2998). The hydrosol was subjected to liquid-liquid extraction, using dichloromethane to extract the organic phase. 3.4214 g of essential oil was obtained from 356.0 g of the aerial parts by steam distillation for 2 h at atmospheric pressure in a stainless-steel pilot apparatus. Steam distillation yielded 0.9611%. Anhydrous sodium sulfate was used to dry the extract, which was stored at 4 °C in the dark until further analysis.

### 2.2 Essential oil analysis (GC-MS)

Chemical profiling of the extract was performed by gas chromatography–mass spectrometry (GC–MS) using a Shimadzu QP 2010 Plus system equipped with a Rtx-5MS fused-silica capillary column (30 m × 0.25 mm ID, 0.25 μm film thickness) and operated in splitless mode. Helium was used as the carrier gas at 1.0 mL/min, with a 1.0 μL injection volume. Mass spectra were acquired in full-scan mode (35–500 m/z) at 1.56scans/s using electron-impact ionization (EI+, 70 eV) with the ion source at 280 °C. The oven temperature was initially held at 50 °C for 5 min, increased at 8 °C/min to 280 °C, and held for 15 min.

Terpenes were identified by comparing retention indices (RI) with those of a homologous series of *n*-alkanes (C7–C40; Supelco) analyzed under identical conditions. Selected compounds were confirmed by co-injection with authentic commercial standards (Sigma; ≥99% purity), whereas α-thujone and β-thujone were tentatively assigned based on comparison of their retention indices and mass spectra with the NIST and Wiley mass spectral libraries (NIST/EPA/NIH Mass Spectral Library, version 2.0 g; NIST data version 17) and with published MS data (Adams et al., 2006; Tuberoso et al., 2005; Oses et al., 2025).

### 2.3 Animals

Experiments were performed in male and female C57BL/6 J mice aged PND 30–45. A total of 25 mice (13 males/12 females) were used across all experiments, and data from both sexes were pooled for electrophysiological analyses. Animals were housed under a 12 h light/dark cycle with *ad libitum* access to food and water. All procedures complied with the NIH Guide for the Care and Use of Laboratory Animals and were approved by the Institutional Animal Ethics Committee of the Universidad de Valparaíso (Bioethics Approval CBC 153–2026).

### 2.4 Acute hippocampal slice preparation

Mice were deeply anesthetized with isoflurane and decapitated. Brains were rapidly removed and immersed in ice-cold artificial cerebrospinal fluid (ACSF) containing (in mM): 124 NaCl, 2.69 kC l, 1.25 KH_2_PO_4_, 1.2 MgSO_4_, 26 NaHCO_3_, 10 glucose, and 2.5 CaCl_2_, continuously bubbled with 95% O_2_ and 5%CO_2_ to maintain pH7.4. Coronal slices containing the dorsal hippocampus (300 μm) were cut with a vibratome and allowed to recover for at least 1 h in oxygenated ACSF at room temperature (20 °C–22 °C). During recordings, slices were transferred to a submerged recording chamber and continuously perfused with ACSF at a rate of 1–2 mL/min (Guiffa-Gómez et al., 2026; Santander et al., 2025; Pérez et al., 2019; Ahumada et al., 2013).

### 2.5 Whole-cell recordings of evoked inhibitory postsynaptic currents

Whole-cell voltage-clamp recordings were obtained from visually identified dorsal CA1 pyramidal neurons using infrared differential interference contrast microscopy. Patch pipettes (4–6 MΩ) were filled with a Cs-based internal solution containing (in mM): 111 Cs-gluconate, 10 HEPES, 10 EGTA, 2 glucose, 1 CaCl_2_, 8 NaCl, 5 ATP-Mg^2+^, and 0.4 GTP-Na^+^, adjusted to pH 7.2–7.3 with CsOH. Cells were accepted with seal resistance >1GΩ and access resistance of 8–20MΩ, with <20% change during recording. Recordings were performed using a Cornerstone PC-ONE amplifier and PC-ONE-20 headstage (Dagan Corporation), with currents low-pass filtered at 3 kHz, digitized at 10 kHz using a Digidata 1440A, and acquired with Clampex 10.7 (Molecular Devices). Capacitive currents were compensated by 70%, and recordings began after a 5–10 min stabilization period. eIPSCs were evoked using a silver bipolar stimulating electrode connected to an isolation unit (Isoflex, AMPI) through a septated glass pipette filled with ACSF. The electrode was positioned in the stratum pyramidale, 50–100 µm from the recorded neuron, to evoke perisomatic eIPSCs (Figures 1A,B). eIPSCs were recorded at 0 mV in the presence of MK-801 (50 µM) and CNQX (20 µM) to isolate inhibitory currents.

**FIGURE 1:**
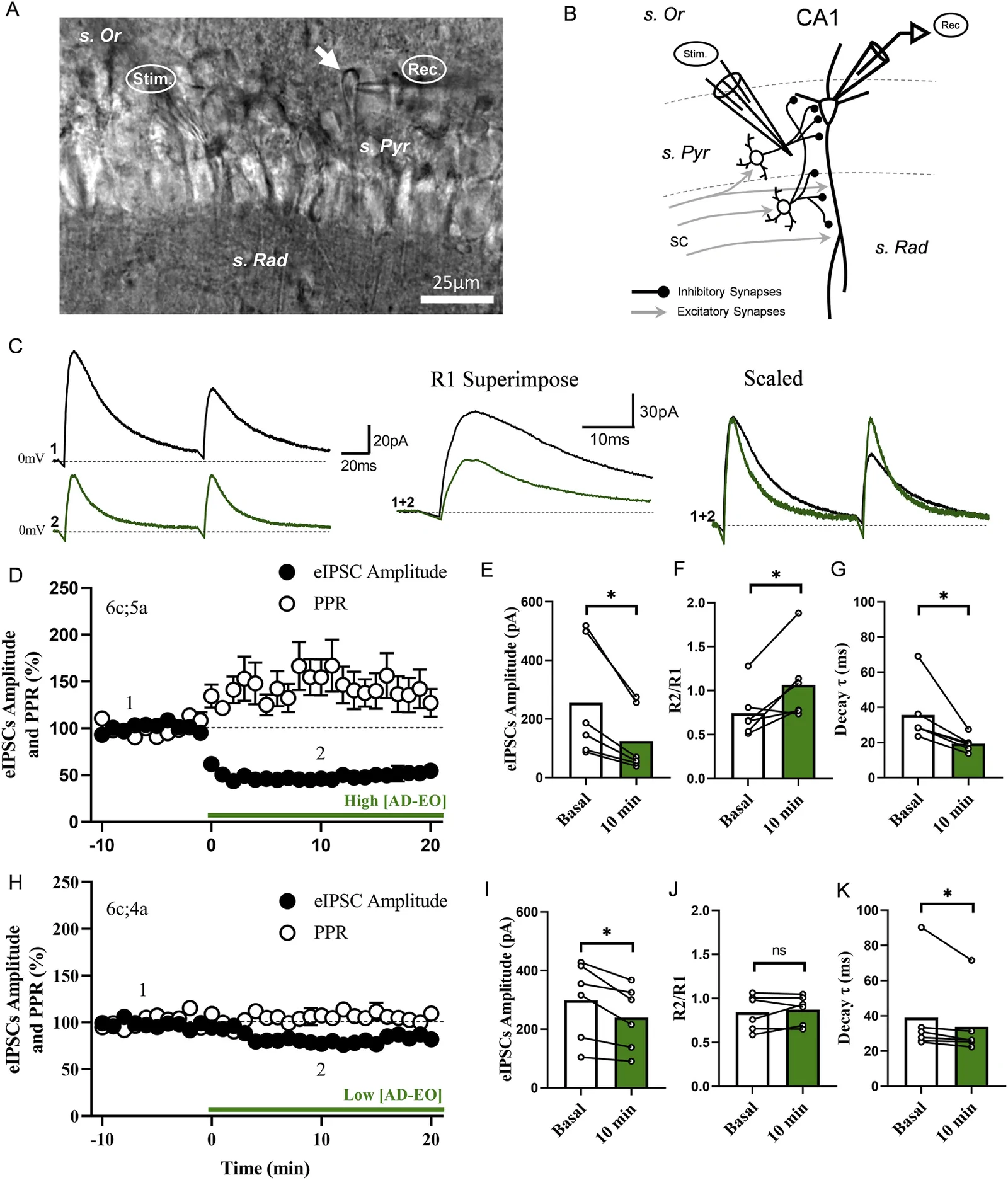
AD-EO depresses perisomatic inhibitory synaptic transmission in CA1 pyramidal neurons. **(A)** Representative IR-DIC image showing the recording configuration in the CA1 region of acute dorsal hippocampal slices, with the recording pipette positioned onto a CA1 pyramidal neuron and the stimulating electrode placed in the stratum pyramidale to evoke perisomatic inhibitory postsynaptic currents. **(B)** Schematic representation of the experimental configuration used to record eIPSCs from CA1 pyramidal neurons during perisomatic stimulation. **(C)** Representative paired-pulse eIPSC traces recorded under basal conditions (black) and after 10 min of AD-EO application (green). Superimposed and scaled traces are shown to compare waveform kinetics independently of peak amplitude. **(D)** Time course of normalized eIPSC amplitude and PPR before and during bath application of the high AD-EO condition (estimated thujone-equivalent concentration: ∼1.13 mM). **(E–G)** Summary plots showing paired comparisons of eIPSC amplitude, PPR, and decay τ under basal conditions and after 10 min of high AD-EO application (∼1.13 mM estimated thujone-equivalent concentration). **(H)** Time course of normalized eIPSC amplitude and PPR before and during bath application of the low AD-EO condition (estimated thujone-equivalent concentration: ∼4.5 µM). **(I–K)** Summary plots showing paired comparisons of eIPSC amplitude, PPR, and decay τ under basal conditions and after 10 min of low AD-EO application (∼4.5 µM estimated thujone-equivalent concentration). Data are presented as mean ± SEM in time-course plots. In paired summary plots, lines represent individual cells and bars represent mean values. Experiments at the high AD-EO condition were performed in 6 cells from 5 animals (6c; 5a), whereas experiments at the low AD-EO condition were performed in 6 cells from 4 animals (6c; 4a). Statistical comparisons between basal and AD-EO conditions were performed using paired Student’s *t*-test or Wilcoxon matched-pairs signed-rank test, according to data normality. *p < 0.05; ns, not significant.

### 2.6 Recording of miniature inhibitory postsynaptic currents

Miniature inhibitory postsynaptic currents (mIPSCs) were recorded in the presence of TTX (500 nM) to block action potential-dependent neurotransmitter release. Recordings were obtained during a 10-min baseline period and following bath application of the intermediate-high AD-EO condition (∼113 µM estimated thujone-equivalent concentration). For the AD-EO condition, the 10–20 min period after the onset of application was analyzed. mIPSCs were analyzed offline using Clampfit (Molecular Devices) with automatic event detection by Template Search, followed by visual inspection and manual curation to exclude false events, noise, and artifacts. Identical detection parameters were applied to both conditions. Mean mIPSC frequency and amplitude were calculated for each 10-min analysis period, and inter-event interval and amplitude distributions were assessed using cumulative probability distributions.

### 2.7 Preparation and bath application of AD-EO

AD-EO was diluted in dimethyl sulfoxide (DMSO) before bath application. Experimental concentrations are reported primarily as nominal AD-EO concentrations in the bath. Because GC-MS analysis showed that α-thujone and β-thujone accounted for 92.44% of the total chromatographic area, estimated thujone-equivalent concentrations were also calculated to facilitate pharmacological interpretation. Based on the total oil mass (3.4214 g), gravimetrically determined density (0.93 g/mL), and thujone molecular weight (152.23 g/mol), the estimated thujone-equivalent concentration in pure AD-EO was ∼5.65 M.

For the high, intermediate-high, and intermediate-low conditions, a 50% (v/v) AD-EO stock solution in DMSO was prepared, corresponding to an estimated thujone-equivalent concentration of approximately 2.83 M. Addition of 10, 1, and 0.5 μL of this stock to 25 mL ACSF yielded final AD-EO concentrations of 0.2, 0.02, and 0.01 μL/mL, corresponding to estimated thujone-equivalent concentrations of ∼1,130, 113 and 56.5 μM, respectively. For the low condition, a 10% (v/v) AD-EO stock solution in DMSO was prepared, and 0.2 µL of this stock was added to 25 mL ACSF, yielding 0.0008 μL/mL (0.8 nL/mL) AD-EO and an estimated thujone-equivalent concentration of ∼4.5 μM. Thus, the experimental conditions are referred to hereafter as high (∼1.13 mM), intermediate-high (∼113 μM), intermediate-low (∼56.5 μM), and low (∼4.5 μM), based on estimated thujone-equivalent concentrations (Supplementary Table S1). Stock solutions were thoroughly vortexed before use. After addition of the corresponding stock aliquot to ACSF, the solution was vigorously mixed by repeated aspiration and dispensing with a Pasteur pipette until visually homogeneous before bath perfusion.

Final bath DMSO concentrations were 0.02%, 0.002%, 0.001%, and 0.00072% (v/v) for the high, intermediate-high, intermediate-low, and low AD-EO conditions, respectively. As a conservative vehicle control, DMSO alone was bath-applied at 0.04% (v/v; 10 µL in 25 mL ACSF), twice the maximum concentration used in the AD-EO experiments (Supplementary Table S1), to assess potential effects on inhibitory synaptic transmission.

### 2.8 Electrophysiological analysis

eIPSC amplitude was quantified from the peak current and expressed as a percentage of baseline, defined as the mean response recorded during the 10 min preceding AD-EO application. Paired-pulse ratio (PPR) was calculated as R_2_/R_1_, where R_1_ and R_2_ correspond to the peak amplitudes of the first and second eIPSCs, respectively, and was normalized to baseline in the same manner as eIPSC amplitude. Paired-pulse stimuli were delivered at 0.1 Hz with a 100 ms interstimulus interval, and responses recorded during baseline and 10 min after AD-EO exposure were used for analysis. Decay time constants (τ) were obtained by fitting a single-exponential function to the decay phase of averaged eIPSC traces generated from the last 5 min of the baseline period and from 10 to 15 min after AD-EO application. Presynaptic contributions were further evaluated by coefficient of variation (CV) analysis using the classical relationship between normalized mean amplitude and 1/CV^2^ (Faber and Korn, 1991; Brock et al., 2020; Guiffa-Gómez et al., 2026). Data are presented as mean ± SEM. Sample sizes are reported as recorded cells and animals in the corresponding figure legends.

### 2.9 Statistical analysis

We assessed normality using the Shapiro–Wilk test. Paired baseline and post-application data were analyzed using a paired t-test or a Wilcoxon signed-rank test, as appropriate. Differences among AD-EO concentrations were assessed using a one-way ANOVA with a Tukey *post hoc* test or a Kruskal–Wallis test with a Dunn *post hoc* test, depending on the data distribution. Washout experiments were analyzed using repeated-measures one-way ANOVA with Tukey’s *post hoc* test. The Kolmogorov–Smirnov test was used to compare cumulative mIPSC inter-event interval and amplitude distributions, and linear regression was used to assess CV relationships. Multiple-comparison corrections were applied to *post hoc* tests. No additional correction was applied to prespecified within-condition baseline-versus-treatment comparisons. Data are presented as mean ± SEM unless otherwise indicated. Sample sizes are reported as recorded cells (c) and contributing animals (a). P < 0.05 was considered significant. Statistical analyses were performed using GraphPad Prism 8.

## 3 Results

### 3.1 High α/β-thujone content defines the AD-EO chemotype

Since the biological activity of essential oils depends on their chemical composition, the essential oil (EO) was characterized by gas GC-MS. Thirteen volatile compounds were detected, the predominant ones being α-thujone (89.06%) and β-thujone (3.38%), which together accounted for 92.44% of the total chromatographic area(Supplementary Figure S1). Other compounds, including eucalyptol (1.15%), 2-hexanone (1.07%), sabinene (0.99%), and terpinen-4-ol (0.39%), were detected at substantially lower levels (see Table 1). The total ion chromatogram further demonstrated the predominance of peaks tentatively assigned to thujone isomers, supporting the classification of AD-EO as a thujone-enriched chemotype and providing a chemical basis for interpreting its electrophysiological effects.

**TABLE 1:** Results of the Gas Chromatography-Mass Spectrometry (GC-MS) analysis of the essential oil of *Aloysia deserticola* Phil.

N°	Tr (min)	RIe	RIt	Relative amount (%)	Compounds	Id.	CAS
1	3.773	788	783	0.68	3-Hexanone	B	589–38–8
2	3.854	791	790	1.07	2-Hexanone	B	591–78–6
3	3.980	795	797	0.31	3-Hexanol	B	623–37–0
4	4.080	799	800	0.61	2-Hexanol	B	626–93–7
5	8.318	949	—	0.97	Alcohol NI	B	—
6	8.579	958	—	0.79	Ketone NI	B	—
7	8.987	973	973	0.99	(+) sabinene	B	3,387–41–5
8	10.267	1,023	1,023	0.37	p-Cymene	A	99–87–6
9	10.428	1,030	1,031	1.15	Eucalyptol	A	470–82–6
10	**12.176**	**1,105**	**1,106**	**89.06**	**α-Thujone**	**B**	**546–80–5**
11	**12.380**	**1,115**	**1,114**	**3.38**	**β-Thujone**	**B**	**471–15–8**
12	13.082	1,149	1,151	0.23	Neo-isothujan-3-ol	B	21653–19–0
13	13.655	1,177	1,177	0.39	Terpinen-4-ol	A	562–74–3

Identification of compounds is based on A = comparison with authentic standards, B = tentative identification based on comparison of retention index and mass spectrum (NIST14 library, similarity > 85%) with literature and database values. RIe: Experimental Kovats retention index calculated relative to a C7–C40 n-alkane standard on an Rtx-5MS, capillary column; RIt, Literature Kovats retention index; NI, Not identified. Compounds with the highest relative abundance in the essential oil of *Aloysia deserticola* Phil. are indicated in bold.

### 3.2 AD-EO depresses inhibitory synaptic currents in dorsal CA1 pyramidal neurons in a concentration-dependent manner

Given the thujone-rich chemotype of AD-EO, we hypothesized that it would reduce inhibitory synaptic transmission in hippocampal circuits. Previous evidence showing that α-thujone can inhibit GABA_A_ receptor (GABA_A_R)-mediated currents through a picrotoxin-like, noncompetitive mechanism led us to this hypothesis (Olsen, 2000; Höld et al., 2000; Szczot et al., 2012; Czyzewska and Mozrzymas, 2013; Pelkonen et al., 2013).

Because AD-EO was applied as serial dilutions of a complex essential oil, each experimental condition was assigned an estimated thujone-equivalent concentration based on its GC–MS composition. Accordingly, the tested conditions are referred to hereafter as high (∼1.13 mM), intermediate-high (∼113 µM), intermediate-low (∼56.5 µM), and low (∼4.5 µM) thujone-equivalent AD-EO conditions, corresponding to progressively more dilute AD-EO preparations (Supplementary Table S1).

Bath application of AD-EO significantly reduced eIPSC amplitude in dorsal CA1 pyramidal neurons at all concentrations tested. At the highest concentration, eIPSC amplitude decreased from 255.1 ± 81.5 to 124.0 ± 44.8 pA 10 min after application (Wilcoxon matched-pairs test, W = −21.0, p = 0.031) (Figures 1C, E), corresponding to 46.4% ± 3.4% of baseline (paired *t*-test, *t* (5) = 11.40, p < 0.0001) (Figures 1D). Similar reductions were observed at intermediate-high (273.1 ± 59.1 to 166.5 ± 23.8 pA; p = 0.043) (Supplementary Figures S2A,C paired t-test), intermediate-low (320.5 ± 61.8 to 188.4 ± 48.7 pA; p = 0.026) (Supplementary Figure 2G paired t-test), and low concentrations (298.8 ± 54.1 to 239.8 ± 44.9 pA; p = 0.017) (Figure 1I, paired t-test). Normalized eIPSC amplitudes were reduced to 56.5% ± 7.8%, 56.6% ± 7.8%, and 80.1% ± 3.2% of baseline, respectively (Figures 1H, 2B, Supplementary Figures S2B,F). The magnitude of inhibition differed significantly across concentrations (one-way ANOVA, F (3,19) = 6.31, p = 0.0037) (Figures 2B), with Tukey’s test showing greater suppression at the highest and intermediate-high concentrations than at the lowest concentration (p = 0.0025 and p = 0.038, respectively) The reduction in eIPSC amplitude at the high AD-EO concentration was accompanied by a decrease in the decay time constant (τ), from 35.7 ± 6.9 ms at baseline to 19.5 ± 1.9 ms after AD-EO application (Figures 1G; Wilcoxon matched-pairs signed-rank test, p = 0.031). At the intermediate-high concentration, decay τ decreased from 37.9 ± 5.8 ms at baseline to 26.4 ± 2.8 ms after AD-EO application (Supplementary Figure S2E; paired Student’s t-test, t(5) = 2.885, p = 0.0344). At the intermediate-low concentration, decay τ decreased from 34.3 ± 5.4 ms to 27.0 ± 4.7 ms (Supplementary Figure S2I; paired Student’s t-test, p = 0.035). Similarly, at the low concentration, decay τ decreased from 38.9 ± 10.4 ms to 33.8 ± 7.4 ms (Figures 1K; Wilcoxon matched-pairs signed-rank test, p = 0.030). To evaluate the potential presynaptic effects of AD-EO, we analyzed the PPR before and after its application. At the high concentration, the PPR increased from 0.74 ± 0.10 at baseline to 1.10 ± 0.20 10 min after the onset of the AD-EO application (Figure 1F; paired t-test, p = 0.032). Normalized PPR increased to 157.1% ± 17.6% of baseline (Figures 1D, 2C; paired t-test, t (5) = 3.4, p = 0.02). At the intermediate-high concentration, the PPR increased from 0.71 ± 0.06 to 0.86 ± 0.05 10 minutes after the onset of the AD-EO application (see Supplementary Figure S2D; paired t-test, t (5) = 3.8, p = 0.012), reaching 120.0% ± 5.5% of the baseline in the normalized analysis (t (5) = 3.4, p = 0.019) (see Supplementary Figures S2B, C). At the intermediate-low concentration, PPR increased from 0.81 ± 0.07 to 1.00 ± 0.06 10 minutes after the onset of AD-EO application (see Supplementary Figure S2H; paired t-test, t (4) = 4.726, p = 0.0091), corresponding to 123.2% ± 8.6% of baseline (see Supplementary Figure S2F and Figure S2C; p = 0.018) (Figure 2C; Supplementary Figure S2F).

**FIGURE 2:**
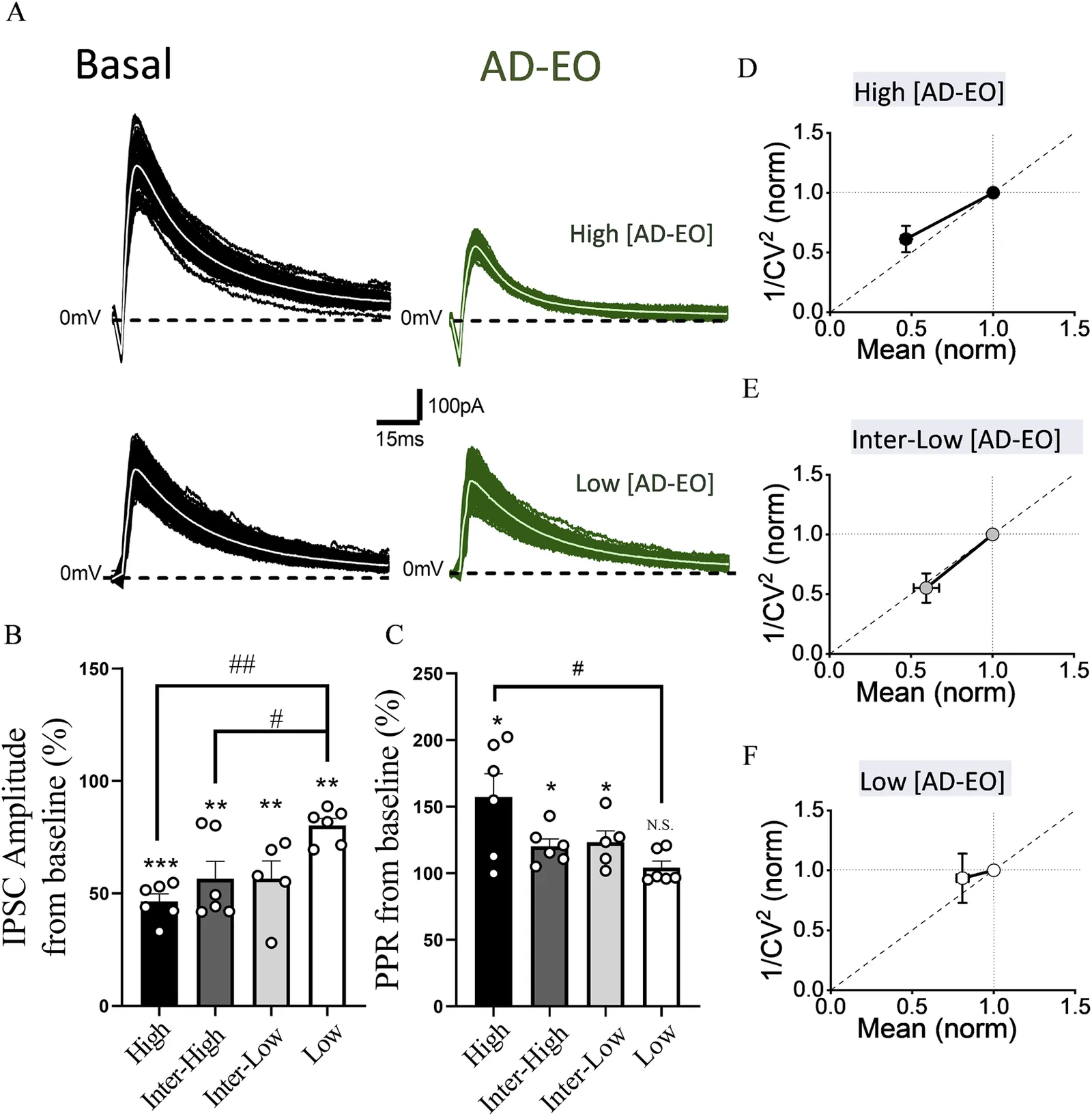
AD-EO produces concentration-dependent changes in eIPSC amplitude, PPR, and CV-based parameters. **(A)** Representative superimposed eIPSC traces recorded under basal conditions and after 10 min of AD-EO application at the high and low AD-EO conditions. Black traces indicate basal responses and green traces indicate responses after 10 min of AD-EO application. **(B)** Summary data of normalized eIPSC amplitude after AD-EO application under the high (∼1.13 mM), intermediate-high (∼113 µM), intermediate-low (∼56.5 µM), and low (∼4.5 µM) AD-EO conditions. Values in parentheses indicate estimated thujone-equivalent concentrations. **(C)** Summary data of normalized PPR after AD-EO application under the same four conditions described in (B). **(D–F)** Coefficient-of-variation-based analysis showing the relationship between normalized mean eIPSC amplitude and normalized 1/CV^2^ after AD-EO application at the same AD-EO conditions described in (B) and (C). Dashed diagonal lines indicate the theoretical prediction for a purely presynaptic change, whereas dotted horizontal and vertical lines indicate the normalized basal value. Sample sizes for the concentration-response analysis were: high, 6 cells from 5 animals (6c; 5a); intermediate-high, 6 cells from 5 animals (6c; 5a); intermediate-low, 5 cells from 4 animals (5c; 4a); and low, 6 cells from 4 animals (6c; 4a). Bars represent mean ± SEM, and individual points correspond to single recorded cells. Asterisks indicate comparisons between baseline and AD-EO conditions, assessed using paired Student’s t-test or Wilcoxon matched-pairs signed-rank test according to data normality. Hash symbols indicate comparisons among AD-EO conditions, assessed using one-way ANOVA followed by Tukey’s *post hoc* test. *p < 0.05, **p < 0.01, ***p < 0.001 versus baseline; #p < 0.05, ##p < 0.01, ###p < 0.001 between indicated conditions.

In contrast, the PPR did not change significantly at low concentrations. It increased from 0.84 ± 0.08 at baseline to 0.87 ± 0.07 10 min after the onset of AD-EO application (Figure 1J; paired t-test, t (5) = 0.79, p = 0.46). Normalized PPR reached 104.1% ± 4.9% of baseline (Figures 1H, 2C; t (5) = 0.32, p = 0.75). Across concentrations, normalized post-application PPR differed significantly (Figure 2C; one-way ANOVA, F (3, 19) = 4.3, p = 0.018). Tukey’s test revealed a higher normalized PPR at the high concentration than at the low concentration (p = 0.01), with no other significant differences. Thus, AD-EO produced a concentration-dependent increase in PPR, which was most pronounced at the highest concentration.

These results suggest that the presynaptic component of AD-EO action is mainly detectable at intermediate-to-high concentrations. As a complementary approach, we applied CV analysis (Figure 2A) to evaluate changes in release probability and synaptic reliability (Faber and Korn, 1991; Brock et al., 2020; Guiffa-Gómez et al., 2026). The regression slopes between normalized amplitude and 1/CV^2^ were statistically significant at the highest tested AD-EO concentration (slope = 0.98 ± 0.29, p = 0.019; Figure 2D) and at the intermediate-low concentration (slope = 1.06 ± 0.25, p = 0.014; Figure 2E). However, they were not significant at the lowest concentration (slope = 0.43 ± 1.40, p = 0.776; Figure 2F). To further assess the effects of AD-EO on inhibitory synaptic transmission, we recorded mIPSCs (Figure 3E). AD-EO significantly reduced mIPSC frequency from 1.17 ± 0.31 Hz at baseline to 0.31 ± 0.11 Hz 10 min after the onset of AD-EO application (Figure 3F; paired t-test, t (4) = 3.8, p = 0.018), accompanied by a shift in the cumulative inter-event interval distribution toward longer intervals (Figure 3H; Kolmogorov–Smirnov test, D = 0.26, p < 0.0001). mIPSC amplitude decreased modestly but significantly from 15.33 ± 0.82 pA to 13.50 ± 0.76 pA (Figure 3G; paired t-test, t (4) = 3.98, p = 0.016), with a corresponding shift in the cumulative amplitude distribution toward lower values (Figure 3I; Kolmogorov–Smirnov test, D = 0.34, p < 0.0001). Together with the PPR and CV analyses, these findings suggest that AD-EO affects both the presynaptic and postsynaptic components of inhibitory synaptic transmission in dorsal CA1 pyramidal neurons.

**FIGURE 3:**
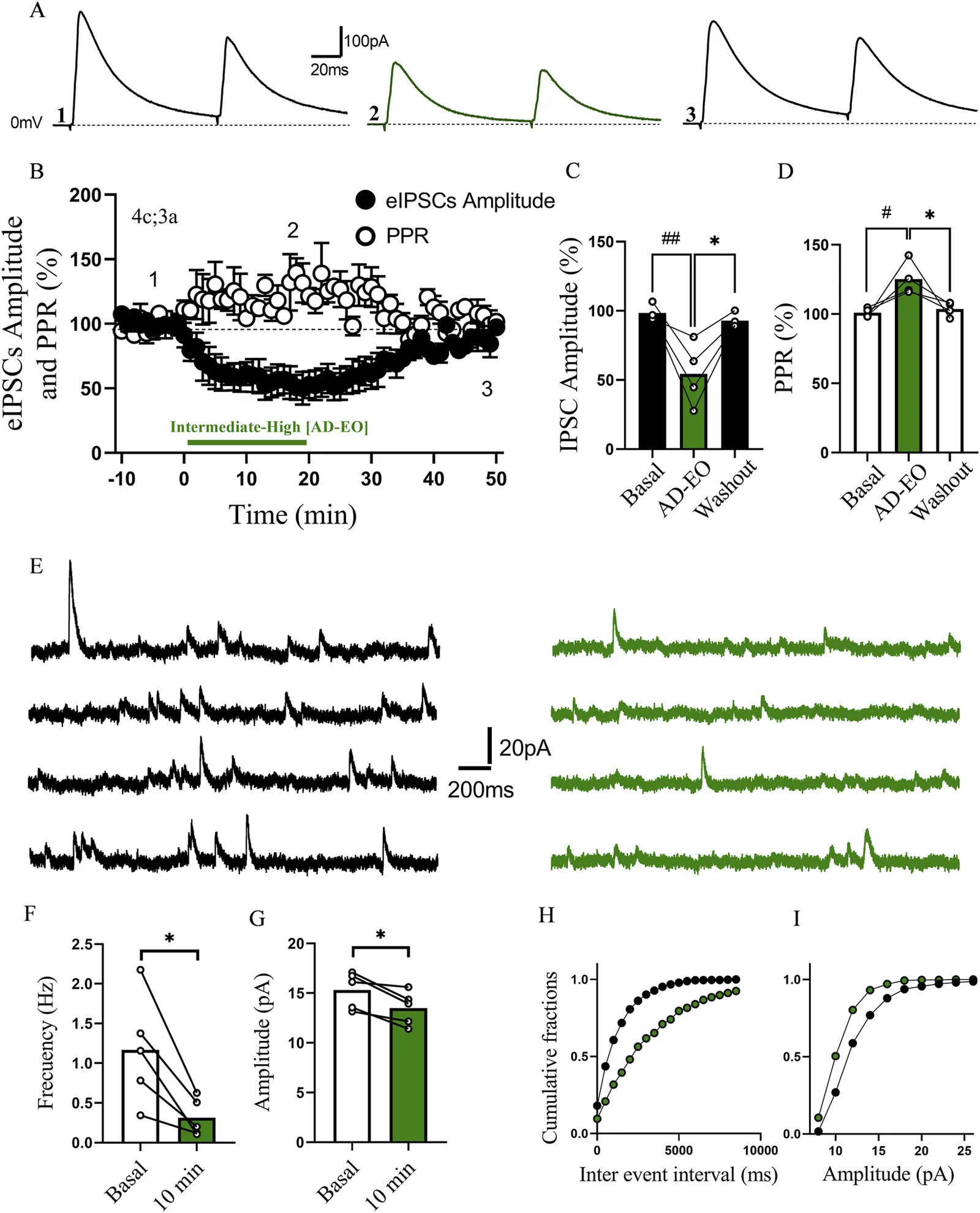
Effects of the intermediate-high AD-EO condition on inhibitory synaptic transmission are reversible upon washout and are accompanied by changes in miniature inhibitory postsynaptic currents. **(A)** Representative paired-pulse eIPSC traces recorded under basal conditions (1), during bath application of the intermediate-high AD-EO condition (2), and following washout (3). Black traces correspond to basal and washout conditions, whereas green traces correspond to recordings obtained in the presence of AD-EO. **(B)** Time course of normalized eIPSC amplitude and PPR before, during, and after bath application of the intermediate-high AD-EO condition. The green bar indicates the period of AD-EO application. **(C)** Summary plot of normalized eIPSC amplitude under basal conditions, during AD-EO application, and following washout. **(D)** Summary plot of normalized PPR under the same experimental conditions. Washout experiments were performed in 4 cells from 3 animals (4c; 3a). Statistical comparisons were performed using repeated-measures one-way ANOVA followed by Tukey’s multiple comparisons test. **(E)** Representative mIPSC recordings obtained from CA1 pyramidal neurons under basal conditions (black) and 10 min after application of the intermediate-high AD-EO condition (green). **(F,G)** Paired summary plots showing mIPSC frequency and amplitude, respectively, under basal conditions and after 10 min of AD-EO application. Individual lines represent paired measurements from the same recorded cell. **(H)** Cumulative distribution of mIPSC inter-event intervals under basal conditions and after AD-EO application. **(I)** Cumulative distribution of mIPSC amplitudes under the same conditions. mIPSC experiments were performed in 5 cells from 2 animals (5c; 2a). Statistical comparisons of mIPSC frequency and amplitude were performed using paired Student’s *t*-tests, whereas cumulative distributions were compared using the Kolmogorov–Smirnov test. Bars represent mean ± SEM. *p < 0.05; #*p* < 0.05; **p < 0.01. In panels **(C,D)**, hash symbols indicate comparisons between the basal and AD-EO conditions, and asterisks indicate comparisons between the AD-EO and washout conditions. In panels **(F,G)**, asterisks indicate comparisons between the basal and AD-EO conditions. For cumulative distribution comparisons, *p* < 0.0001.

To evaluate the reversibility of the effects of AD-EO on inhibitory transmission, the amplitude of eIPSCs and the PPR were measured during the washout period. Normalized eIPSC amplitude decreased from 98.5% ± 2.7% at baseline to 54.4% ± 11.6% during AD-EO application. It subsequently recovered to 92.8% ± 2.6% following washout (Figures 3A–D; repeated-measures one-way ANOVA, F (2, 6) = 13.22, p = 0.0063). Tukey’s test revealed significant differences between baseline and AD-EO (p = 0.008) and between AD-EO and washout (p = 0.014), though no significant difference was observed between baseline and washout (p = 0.85). Similarly, normalized PPR increased from 101.1% ± 1.6% at baseline to 125.3% ± 6.1% during AD-EO application, subsequently recovering to 103.7% ± 2.6% after washout (Figure 3D; repeated-measures one-way ANOVA, F (2, 6) = 8.12, p = 0.019). Tukey’s test revealed significant differences between the baseline and the AD-EO (p = 0.024) conditions, as well as between the AD-EO and the washout (p = 0.039) conditions. However, there were no significant differences between the baseline and the washout conditions (p = 0.92). Thus, the effects of AD-EO on eIPSC amplitude and PPR were reversible following washout. To exclude potential vehicle effects, dimethyl sulfoxide (DMSO) was tested at 0.04% (v/v), which is twice the maximum concentration of DMSO present in the AD-EO experimental conditions. DMSO did not alter the eIPSC amplitude, which was 467.9 ± 114.8 pA at baseline and 479.7 ± 92.3 pA 10 min after the onset of DMSO application (see Supplementary Figures S3A–S3C; paired Student's t-test, t (5) = 0.353, p = 0.74). Similarly, PPR and decay time constant remained unchanged from baseline: PPR, from 0.81 ± 0.049 to 0.81 ± 0.033 (Supplementary Figure S3D, paired Student's t-test, t (5) = 0.078, p = 0.94); and decay time constant, from 28.47 ± 4.36 ms to 28.79 ± 3.93 ms (Supplementary Figure S3E, paired Student's t-test, t (5) = 0.194, p = 0.85). To confirm GABA_A_R mediation pharmacologically, picrotoxin (PTX, 50 µM) was applied at the end of a subset of DMSO-control recordings. PTX produced a significant decrease in normalized eIPSC amplitude, dropping from 98.31% ± 1.02% before PTX application to 6.47% ± 1.37% in the presence of PTX (see Supplementary Figures S3F–S3H; paired Student's t-test, t (2) = 50.25, p = 0.0004). These results confirm that the evoked inhibitory currents recorded under our experimental conditions were largely GABA_A_R mediated.

## 4 Discussion

This study provides electrophysiological evidence that acute administration of AD-EO suppresses GABAergic inhibitory synaptic transmission in dorsal hippocampal CA1 pyramidal neurons. Gas chromatography–mass spectrometry (GC–MS) analysis revealed a thujone-rich chemical profile, with peaks tentatively assigned to α- and β-thujone accounting for 92.44% of the total chromatographic area. Consistent with this profile, bath application of AD-EO produced a concentration-dependent reduction in eIPSC amplitude, accompanied by faster decay kinetics. This effect was reversible following washout, which supports the idea that it is an acute and reversible pharmacological effect rather than simple recording rundown. DMSO vehicle at twice the maximum concentration produced no comparable changes in amplitude, PPR, or kinetics. Meanwhile, picrotoxin markedly suppressed the recorded currents, confirming that the responses were predominantly GABA_A_R-mediated.

The data support a model in which AD-EO suppresses inhibitory transmission via combined postsynaptic and presynaptic mechanisms (Olsen, 2000; Höld et al., 2000; Szczot et al., 2012; Czyzewska and Mozrzymas, 2013; Pelkonen et al., 2013). The reduction in eIPSC amplitude, together with the shortened decay kinetics, is consistent with altered postsynaptic GABA_A_R signaling. This finding agrees with prior work showing that α-thujone directly inhibits GABA_A_R-mediated currents. The mIPSC experiments strengthen this interpretation. AD-EO reduced mIPSC amplitude and shifted the cumulative amplitude distribution toward smaller events, even when action-potential-dependent release was blocked by TTX. This is consistent with a postsynaptic contribution that is independent of presynaptic firing.

At the same time, several findings are consistent with an additional presynaptic locus. AD-EO increased PPR at intermediate-to-high concentrations, and CV analysis revealed significant relationships between normalized amplitude and 1/CV^2^ under certain conditions. Furthermore, in the presence of TTX, AD-EO reduced mIPSC frequency and shifted inter-event intervals toward longer durations. This pattern is consistent with either altered spontaneous vesicle fusion probability or reduced numbers of functional release sites. Together, the eIPSC, PPR, CV, and mIPSC data suggest coordinated presynaptic and postsynaptic modulation. However, these approaches cannot independently determine the precise molecular site of action. Direct recordings of GABA_A_R current, responses to exogenous GABA, or isolated presynaptic terminal preparations are needed to further clarify this.

An important caveat concerns the relationship between the observed effects and the chemical composition of AD-EO. Since a whole essential oil, rather than purified α- or β-thujone, was tested, the data cannot confirm that thujone alone is responsible for the observed effects. Minor constituents may contribute additively, synergistically, or antagonistically (Bakkali et al., 2008). Similarly, thujone-equivalent concentrations were estimated from GC-MS peak area and oil density rather than from compound-specific calibration. Therefore, they should be treated as approximate, particularly the highest tested condition (∼1.13 mM), which likely exceeds concentrations expected under conventional physiological exposure. Thus, the tested concentration range represents a pharmacological probe rather than a direct model of *in vivo* exposure following traditional consumption.

The reversibility of AD-EO effects, with eIPSC amplitude and PPR returning to baseline levels after washout, suggests transient pharmacological modulation rather than permanent synaptic disruption. However, this does not rule out toxicity or longer-lasting effects *in vivo*, particularly with high exposure. Functionally, the suppression of CA1 GABAergic inhibition could shift the balance of excitation and inhibition and alter the activity of the hippocampal network, though the effects on excitability, plasticity, and behavior were not directly tested here. These findings raise an apparent tension with prior reports of the anxiolytic- and antidepressant-like effects of AD-EO (Benites et al., 2013). Behavioral outcomes from complex essential oils may depend on the dose, route, duration, and brain region targeted, while reduced inhibition could, conversely, predispose one to proconvulsant effects at sufficiently high exposure. Therefore, these findings should not be interpreted as evidence that reduced hippocampal inhibition is inherently beneficial.

The study has several limitations. First, reliance on acute slices limits extrapolation to intact-brain pharmacokinetics and metabolism. Second, pooling male and female animals without the power to assess sex differences was done. Third, whole oil was tested rather than isolated constituents. To clarify compound specificity and the functional and therapeutic relevance of these effects, future studies must incorporate purified thujone isomers, calibrated quantification, and *in vivo* behavioral and electrophysiological approaches.

## Data Availability

The raw data supporting the conclusions of this article will be made available by the authors, without undue reservation.

## Supplementary material

SUPPLEMENTARY FIGURE S1 Representative GC-MS total ion chromatogram (TIC) of the essential oil obtained from *Aloysia deserticola* Phil. **(A)** The chromatographic profile displays the predominance of thujone isomers and other minor constituents. Numbered peaks correspond to the compounds identified in Table 1: (1) 3-hexanone, (2) 2-hexanone, (3) 3-hexanol, (4) 2-hexanol, (5) unidentified alcohol, (6) unidentified ketone, (7) (+)-sabinene, (8) p-cymene, (9) eucalyptol, (10) α-thujone, (11) β-thujone, (12) neo-isothujan-3-ol, and (13) terpinen-4-ol. Chemical structures of the two thujone isomers are shown above their respective signals.

SUPPLEMENTARY FIGURE S2 Effects of intermediate-high and intermediate-low AD-EO conditions on perisomatic inhibitory synaptic transmission in CA1 pyramidal neurons. **(A)** Representative paired-pulse eIPSC traces recorded under basal conditions and after 10 min of bath application of the intermediate-high AD-EO condition (estimated thujone-equivalent concentration: ∼113 µM). Black traces correspond to basal recordings and green traces to recordings obtained after AD-EO application. Superimposed and scaled traces are shown to compare waveform kinetics independently of peak amplitude. **(B)** Time course of normalized eIPSC amplitude and PPR before and during bath application of the intermediate-high AD-EO condition. **(C–E)** Summary plots showing paired comparisons of eIPSC amplitude, PPR, and decay τ, respectively, under basal conditions and after 10 min of intermediate-high AD-EO application. Experiments were performed in 6 cells from 5 animals (6c; 5a). **(F)** Time course of normalized eIPSC amplitude and PPR before and during bath application of the intermediate-low AD-EO condition (estimated thujone-equivalent concentration: ∼56.5 µM). **(G–I)** Summary plots showing paired comparisons of eIPSC amplitude, PPR, and decay τ, respectively, under basal conditions and after 10 min of intermediate-low AD-EO application. Experiments were performed in 5 cells from 4 animals (5c; 4a). Time-course data are expressed as mean ± SEM. In paired summary plots, lines represent individual recorded cells and bars represent mean values. Statistical comparisons between basal and AD-EO conditions were performed using paired Student’s *t*-tests. * *p*<0.05.

SUPPLEMENTARY FIGURE S3 DMSO vehicle does not affect perisomatic inhibitory synaptic transmission, and picrotoxin confirms GABA_A_ receptor-mediated eIPSCs in CA1 pyramidal neurons. **(A)** Representative paired-pulse eIPSC traces recorded under basal conditions and after 10 min of bath application of DMSO (0.04% v/v; 10 µL in 25 mL ACSF). Black traces correspond to basal recordings and blue traces to recordings obtained after DMSO application. Superimposed and scaled traces are shown to compare waveform kinetics independently of peak amplitude. **(B)** Time course of normalized eIPSC amplitude and PPR before and during bath application of DMSO. **(C–E)** Summary plots showing paired comparisons of eIPSC amplitude, PPR, and decay τ under basal conditions and after 10 min of DMSO application. **(F)** Time course of normalized eIPSC amplitude before and during bath application of picrotoxin (PTX, 50 µM) in a subset of the DMSO-control recordings (3 cells). **(G)** Paired summary plot showing normalized eIPSC amplitude before and during PTX application. **(H)** Representative eIPSC traces recorded before and during PTX application, illustrating the marked suppression of the evoked inhibitory current. Time-course data are expressed as mean ± SEM. In paired summary plots, lines represent individual cells and bars represent mean values. DMSO-control experiments were performed in 6 cells from 4 animals (6c; 4a), and PTX was subsequently applied in a subset of these recordings (3 cells). Statistical comparisons between baseline and DMSO conditions and between pre-PTX and PTX conditions were performed using paired Student’s t-tests. ns, not significant; *p* < 0.001.

SUPPLEMENTARY TABLE S1 Preparation and final bath concentrations of AD-EO experimental solutions. AD-EO was diluted in DMSO to generate the indicated stock solutions and subsequently added to 25 mL of ACSF to obtain the four experimental conditions: high, intermediate-high, intermediate-low, and low. Final nominal AD-EO concentrations are reported as µL/mL and % (v/v), together with the corresponding final DMSO concentration. Estimated thujone-equivalent concentrations were calculated from the AD-EO volume added, the experimentally determined oil density, and the combined relative chromatographic area of the peaks tentatively assigned to α- and β-thujone. These values should therefore be interpreted as approximate thujone-equivalent concentrations rather than absolute thujone concentrations. The DMSO vehicle control contained 0.040% (v/v) DMSO, corresponding to twice the maximum DMSO concentration present in the AD-EO experimental conditions.
