# Supplementary material for: Aloysia deserticola (rica-rica) essential oil impairs inhibitory synaptic transmission in hippocampal CA1 pyramidal neurons
Source: Front Pharmacol. 2026 Oct 14;17:1917739. doi: 10.3389/fphar.2026.1917739 (PMC13713359; doi:10.3389/fphar.2026.1917739)
Supplement: Supplementary file 1 [file Table1.doc]

**Supplementary Table 1:** Preparation and final bath concentrations of AD-EO experimental solutions.

| **Experimental condition** | **Stock solution** | **Stock volume added to 25 mL ACSF (µL)** | **AD-EO volume added (µL)** | **Final nominal AD-EO concentration (µL/mL)** | **Final AD-EO (% v/v)** | **Final DMSO (% v/v)** | **Estimated thujone concentration (µM)** |
| --- | --- | --- | --- | --- | --- | --- | --- |
| **High** | 50% AD-EO / 50% DMSO (v/v) | 10 | 5 | 0.20 | 0.020 | 0.020 | ~1130 |
| **Intermediate-high** | 50% AD-EO / 50% DMSO (v/v) | 1 | 0.5 | 0.020 | 0.0020 | 0.0020 | ~113 |
| **Intermediate-low** | 50% AD-EO / 50% DMSO (v/v) | 0.5 | 0.25 | 0.010 | 0.0010 | 0.0010 | ~56.5 |
| **Low** | 10% AD-EO / 90% DMSO (v/v) | 0.2 | 0.02 | 0.0008 | 0.000080 | 0.00072 | ~4.5 |
| **DMSO vehicle control** | 100% DMSO | 10 | 0 | 0 | 0 | 0.040 | 0 |
